# Tuning Poly(dimethylsiloxane) Hydrophilization and Coating Stability via the Optimization of Polyethylene Glycol Molecular Weight

**DOI:** 10.3390/polym17243296

**Published:** 2025-12-12

**Authors:** Daniil Golubchikov, Konstantin Oleynichenko, Anton Murashko, Yuri Efremov, Sofia Safaryan, Frederico D. A. S. Pereira, Galina Nifontova, Anna Solovieva, Anastasia Shpichka, Peter Timashev

**Affiliations:** 1 Institute for Regenerative Medicine, Sechenov University, 119991 Moscow, Russia; 2 Department of Chemistry, Lomonosov Moscow State University, 119991 Moscow, Russia; 3 N.N. Semenov Federal Research Center for Chemical Physics, Russian Academy of Sciences, 119334 Moscow, Russia

**Keywords:** polydimethylsiloxane, polyethylene glycol, surface modification, microfluidics, hydrophilic coating, antibiofouling

## Abstract

Polydimethylsiloxane (PDMS) is widely used in microfluidics and medical devices; however, its inherent hydrophobicity limits its applications. This can be resolved by the formation of polyethylene glycol (PEG)-based hydrophilic coatings. Here, we aimed to prove that PDMS surfaces modified with low molecular weight PEG (400) provided a more stable hydrophilic surface. The lowest contact angle achieved via using PEG400 and the “grafting from” approach was 8.6 ± 3.5°. Under perfusion conditions, imitating arterial and capillary flows, such coatings were considerably stable, and the contact angle was kept at 45.5° after 3 days. Moreover, the applied surface modifications preserved surface roughness, elastic modulus, and optical transparency. Thus, these findings confirmed that the “grafting from” approach with low molecular weight PEG could be the most effective strategy to form hydrophilic PDMS coatings with optimal performance in biomedical applications.

## 1. Introduction

Due to its properties (biocompatibility, good mechanical properties, durability, gas permeability, etc. [1]), polydimethylsiloxane (PDMS) is one of the most widespread materials for biomedical applications, including manufacturing various devices (“lab-on-a-chip” (LOC), catheters, implants, tubing, and biosensors [2,3,4]). However, its main disadvantage is hydrophobicity, which is crucial, for instance, in blood contacting devices [5] and affects analyte transport, reduces separation performance and detection sensitivity, and can even lead to biomaterial-related thrombosis [1,6]. While applied in LOCs, PDMS hydrophobicity causes difficulties in filling channels with polar liquids mainly used in microphysiological systems [7,8] and increases microchannel flow resistance [8]. Hence, PDMS surface hydrophilicity can be a favorable feature.

One of the approaches to increase it is to modify a surface by changing its chemical composition and structure. For instance, the outer PDMS layer can be oxidized, e.g., via corona discharge [9], ultraviolet light [10], or oxygen plasma treatment [11]. Compared to the first ones, the latter remains the most preferable method due to its swiftness and simplicity [12]. Special attention is paid to plasma interaction with a polymer surface after treating (Toth et al. showed an increase in oxygen content after plasma exposure [13]). The formation of a silica-like layer (less than 10 nm) on the surface is claimed to be the main mechanism of plasma effects, which is confirmed by a number of studies via XPS and contact angle measurements [14,15,16]. Moreover, plasma treatment provides hydroxyl groups on a surface, which can be applied for hydrophilic molecules’ attachment such as polyethylene glycol (PEG) derivatives, zwitterionic molecules, and methacrylates [2,17,18].

PEG is a biocompatible hydrophilic polymer that is widely used to modify PDMS surface [2,8,19]. PEGylation ensures a long-term stable hydrophilic coating due to siloxy groups and PDMS backbone bonding [20] and avoiding non-specific protein binding, crucial for biomedical devices [21]. Additionally, varying the chain length of zwitterionic PEG derivatives can decrease the level of protein adsorption [22].

**Figure 1:**
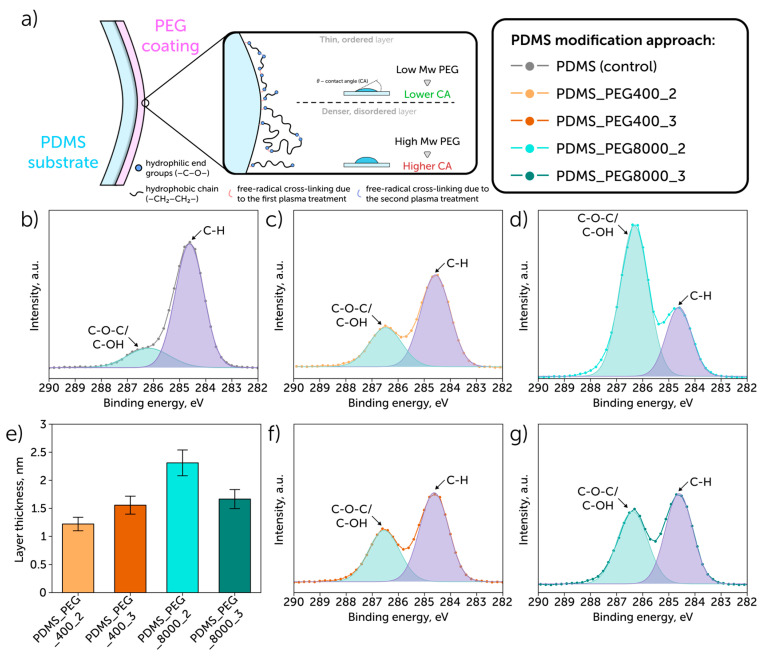
PDMS surface modification: (**a**) scheme of PDMS surface modification with PEG; (**b**–**d**,**f**,**g**) XPS scans of C1s for non-treated PDMS and samples modified with PEG with two molecular weights (400 (**c**,**f**) and 8000 Da (**d**,**g**)) via two approaches (“2-stage grafting” (grafting to—**c**,**d**) and “3-stage grafting” (grafting from—**f**,**g**)); represent the growth of the PEG layer thickness (**e**) with the addition of PEG with a higher molecular weight.

There were several studies focused on PEG grafting on the PDMS surface, aimed at the formation of a hydrophilic layer; however, we revealed that none of those studies provided any dynamic stability of coatings with PEG of different molecular weights, which is important for assessing the applicability of these coatings for microfluidic purposes [23,24,25]. Based on the literature analysis, we revealed that low molecular weight PEG may be more favorable for forming stable hydrophilic coatings [23,25]. Therefore, this study provides a novel hypothesis regarding the beneficial performance of low molecular weight PEG, so we conducted a comparison of low (400 Da) and medium molecular weight polymers (8000 Da), including studies of stability under perfusion conditions. We offered two modification methods: “grafting to”, which included a one-step plasma treatment to graft PEG chains to a PDMS surface (Figure 1a—steps 1 and 2), and “grafting from”, which had the second plasma treatment (Figure 1a—step 3) to cross-link additionally PEG brushes and form a denser layer (that is supposed to be more beneficial for forming more durable coatings).

## 2. Materials and Methods

### 2.1. Materials

Two-component PDMS Redalid 184 (elastomer and curing agent kit) used in this research was obtained from Redalid (Moscow, Russia). The Shore hardness of this specification is 43. PEG with two molecular weights of 400 and 8000 Da was obtained from Panreac (Barcelona, Spain) and Servicebio (Wuhan, China), respectively.

### 2.2. PDMS Sample Preparation

PDMS samples were formed by mixing the elastomer and its curing agent in a mass ratio of 10:1 with subsequent vigorous stirring. To remove captured air bubbles from PDMS, the mixture was degassed under vacuum. Then we poured it into 3D-printed molds to obtain 8 mm diameter disks and cured them at 60 °C for 12 h. To perform bulk modification, we introduced low molecular weight PEG to the mixture.

### 2.3. PEG-Based Surface Modification

We used two approaches to form coatings for the PDMS samples: the “grafting to,” which is generally referred to as the attachment of pre-polymer chains with a reactive end group to a functionalized surface, and “grafting from,” which is the formation of polymer chains on the surface, modified with monomers. The “grafting to” method was modified: PEG chains were attached to the PDMS surface via the plasma treatment, which activates the PEG end groups by the formation of –O•. The PDMS plates were treated with plasma in the system CUTE-1MPR (Femto Science Inc., Hwaseong, Korea) at a frequency of 40 kHz and a power of 50 W for 60 s using synthetic air as a working gas (15 Pa). Then the plates’ surfaces were covered with PEG of two molecular weights of 400 and 8000 Da via the spin-coating method (2-step program: 250 rpm for 15 s, 800 rpm for 20 s). After 10 min, samples were rinsed with deionized water and dried overnight at room temperature. “Grafting from” approach was also modified as follows: firstly, attached PEG chains (as described above for the “grafting to” method) served as monomers, which were further cross-linked to form a more stable and homogeneous layer. To perform the “grafting from” approach, PEGylated PDMS plates underwent an additional treatment with plasma under the same conditions. Then the samples were rinsed with distilled water to remove non-bound PEG and dried overnight at room temperature.

### 2.4. Hydrophilization Retainment Performance

PEG-modified samples were tested under static and dynamic conditions. In the first case, PEG-coated PDMS plates were stored at 25 °C, while the contact angle was measured over a period of 4 weeks. In the second case, we fabricated a microfluidic device from polymethylmethacrylate using CNC machining (List CNC 4060, Jinan, China)—a single-channel chip with 8 mm in diameter and 1 mm in depth cavities. Samples were loaded into the fabricated cavities, and the device was encapsulated to prevent any leakage. Under the pressure applied by a peristaltic pump, distilled water circulated in the chip at flow rates of 0.08 cm/s and 4 cm/s. To estimate the contact angle, the PDMS disks were removed from the chip and dried at room temperature for 2 h. Contact angle values were estimated via a sessile drop method using the device Acam-MSC01 (Apex Instruments, Kolkata, India). Distilled water was used as a testing liquid; each sample was measured at least three times.

### 2.5. Surface Characterization

X-ray photoelectron spectroscopy (XPS) analysis was carried out using a photoelectron spectrometer PHI5000VersaProbeII (Ulvac-Phi Inc., Hagisono, Japan). We obtained concentrations by the method of comparative elemental sensitivity factors using integral intensities of the following lines: C 1s, O 1s, Si 2s, and Na 1s. The elements’ chemical state was determined using high-resolution spectra (HR) recorded at an analyzer transmission energy of 23.5 eV with a step of 0.2 eV. The calibration of the binding energy scale, Eb, was carried out using Au4f—83.96 eV and Cu2p3—932.62 eV. The calculations of layer thickness were performed using the IMFP reference values from previous research [26]. To measure FTIR spectra, we used the spectrometer Spectrum 2 (Perkin Elmer, Shelton, CT, USA) in ATR mode at a wavenumber range of 4000–500 cm^−1^. Atomic force microscopy (AFM) was performed using a microscope Bioscope Resolve (Bruker, Billerica, MA, USA) integrated with an inverted optical microscope Axio Observer (Carl Zeiss, Oberkochen, Germany). For topography and roughness analysis, we applied ScanAsyst Air cantilevers (Bruker, Billerica, MA, USA) with a nominal spring constant of 0.4 N m^−1^ and a nominal tip radius of 2 nm. Scanning was performed in air using PeakForce QNM mode with 10 × 10 μm scans acquired at 512 × 512 resolution, a 2 kHz oscillation frequency, and a 150 nm amplitude with a set point of 5 nN. The acquired topography images were processed (flattened), and roughness parameters—mean roughness (Sa) and root mean square roughness (Sq)—were estimated using Gwyddion software (Version 3.6) [27]. For mechanical analysis, we used TAP525 cantilevers (Bruker, USA) with a nominal spring constant of 200 N m^−1^ (actual: 230 N m^−1^) and a nominal tip radius of 8 nm (actual: 30 nm). Force volume mapping was performed over 10 × 10 μm areas with 40 × 40 force curve arrays, recording 5–6 maps per sample. Each force curve was analyzed to extract Young’s modulus using Hertz’s model implemented with a custom MATLAB code (Version 1.1) [28]. Optical transparency of the samples was investigated using a spectrophotometer Cary 60 UV–Vis (Agilent Technologies, Santa Clara, CA, USA) within a wavelength range of 400–600 nm.

### 2.6. Protein Adsorption

Bovine serum albumin (BSA) (Merck, Rahway, NJ, USA) was labeled using fluorescein isothiocyanate isomer I (FITC, Merck, Rahway, NJ, USA). Briefly, 50 µL of FITC solution (1 mg/mL) in dimethyl sulfoxide (DMSO) was added dropwise to 1 mL of 2 mg/mL BSA in 0.1M bicarbonate buffer (pH 9.2). The reaction mixture was incubated at room temperature in the dark under constant mixing for 2 h. The excess of uncoupled FITC was removed using pre-packed Sephadex G-25 gel filtration columns (GE Healthcare, Chicago, IL, USA). BSA-FITC elution was performed using 0.01 M PBS buffer (pH 7.4). The labeling degree was analyzed spectrophotometrically at 280 nm and 495 nm. The F/P ratio was determined as 0.98–1.00 for the prepared BSA-FITC conjugate.

To assess protein adsorption, 50 µL of 0.5 mg/mL BSA-FITC conjugate solution was placed on the surface of the testing plates and incubated in the dark for 2 h with a subsequent rinsing with PBS to remove unabsorbed protein. Protein adsorption visualization was performed using the imaging system EVOS M5000 (Thermo Fisher Scientific, Waltham, MA, USA). The quantification of the adsorption was performed by calculating the relative surface area of the BSA-FITC conjugate distribution on the samples’ surface. Image processing was executed using ImageJ software (1.54g version) via the Analyze Particles function. All experiments were performed in triplicate (n = 3). All quantitative data were analyzed using one-way ANOVA analysis using Microsoft Excel (Analysis ToolPak Add-in). The differences between groups with * *p* < 0.05 were regarded as statistically significant. The *p*-values are given as the following: ns—not significant; *—*p* < 0.05; and **—*p* < 0.01.

## 3. Results

To reveal changes in the samples’ surface chemistry while grafting PEG with different molecular weights on a PDMS surface, we used XPS. The survey spectra are shown in Figure S1. The surface layer’s elemental composition is presented in Table 1. The concentrations of carbon and oxygen were attributed to the carbon and oxygen of both the PDMS and PEG coatings. The presence of silicon was attributed to the substrate (PDMS). C1s (Figure 1b–d,f,g), Si2p (Figure S2a), and O1s (Figure S2b) spectra were obtained to understand differences among the used modification approaches. Reference values of E_b_ for PDMS are Si 2p3/2—101.8 eV, and C 1s—284.4 eV, O 1s—532.0 eV [29,30]. In the non-modified PDMS sample, we observed a slight excess of O 1s—532.5 eV and C 1s—284.4 eV that can be explained by the PDMS peak overlap with the peak of adsorbed CH–, which is located at 284.8–285.0 eV. The excess of Eb O1s is associated with the PDMS oxidation that follows from the spectrum of silicon Si 2p, which, in addition to the doublet 1-1’ from PDMS, contains a doublet of 103.1 eV. Peak 2 of the C 1s spectrum, which appeared due to C–O–C/C–OH bonds, was considered a coating indicator. This peak is also present (17%) in the initial sample, which is typical for adsorbed hydrocarbons. The peak’s proportion rises with the increase in the PEG molecular weight from 34% (PDMS_PEG400_2) to 74% (PDMS_PEG8000_2), which correlates with the C/Si ratio. We also estimated the PEG coating layer thickness from the XPS data (Figure 1e). The maximum value was observed for PDMS_PEG8000_2 (2.31 nm), while the difference between the two samples modified with PEG400 was negligible. Moreover, the obtained FTIR spectra may act as an additional characterization tool showing slightly more intense absorption lines related to ν(C–O–C)—1150 cm^−1^ and ν(–O–CH_2_–)—2950–2920 cm^−1^ (Figure S3), which can be attributed to the presence of PEG chains on a PDMS surface [31].

**Figure 2:**
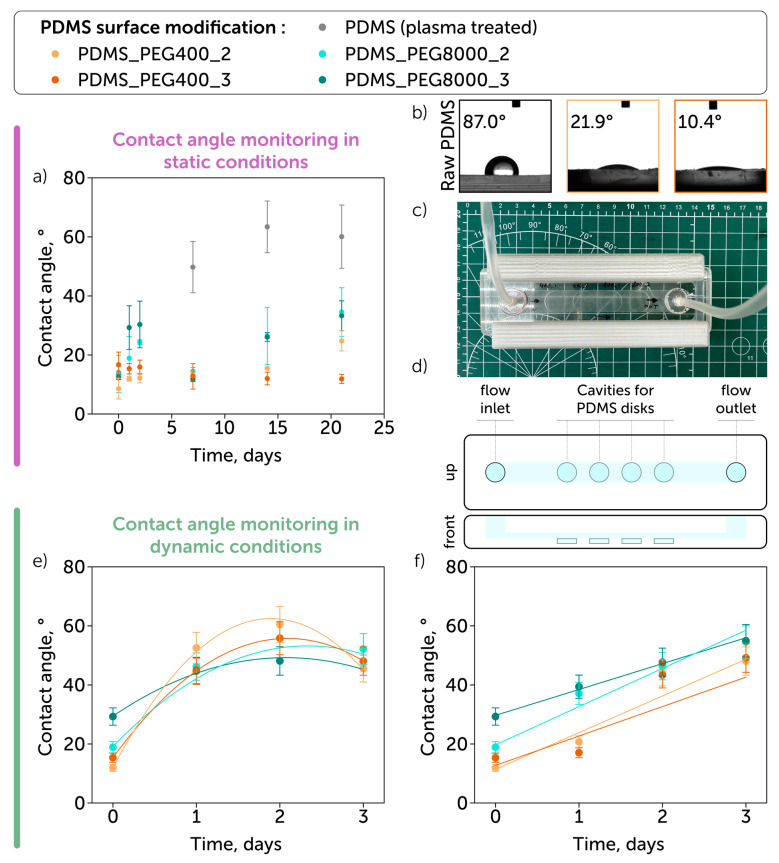
Hydrophilicity of PEG-modified PDMS: (**a**,**b**) static conditions (without flow): contact angle after surface modification; (**c**,**d**) photo and scheme of a microfluidic system implemented; (**e**) dynamic conditions (0.08 cm/s—capillary flow): contact angle after surface modification; (**f**) dynamic conditions (4 cm/s—arterial flow): contact angle after surface modification.

The hydrophilicity of the modified samples was assessed in two experiments: under static and dynamic (in a microfluidic chip) conditions. Figure 2a,b shows the changes in the contact angle values under static conditions during a 3-week period. Initially, all four treatment approaches demonstrated that the contact angle values ranged from 10 to 20° (vs. 97.9° ± 6.9° for the non-modified PDMS). Plasma-treated PDMS used as a control recovered the contact angle to 60° in two weeks, while the grafting (“grafting from”) of low molecular weight PEG retained the initial values until Day 21. The “grafting to” approach with a low molecular weight PEG led to a slightly increased contact angle (20°), while the implementation of high molecular weight PEG was less efficient, leading to contact angle values in the 35–40° range.

To perform experiments under dynamic conditions, we fabricated a one-channel microfluidic chamber (Figure 2c) containing four cavities in the central channel to place samples (Figure 2d). Under the low flow rate conditions (0.08 cm/s, capillary flow model), the contact angle values were retained in 3 days and reached 40–60°; they showed no significant difference between samples treated with low and high molecular weight PEG (Figure 2e). The contact angle dependence on time under flow conditions can be approximated by the second-order polynomial function with parameters presented in Table S1. When the high flow rate (4 cm/s, arterial flow model) was used, the PDMS samples treated with low molecular weight PEG maintained the contact angle value of 20° on Day 1 compared to those treated with high molecular weight PEG (Figure 2f). The contact angle dependence on time under such flow conditions was close to linear (parameters provided in Table S2).

**Figure 3:**
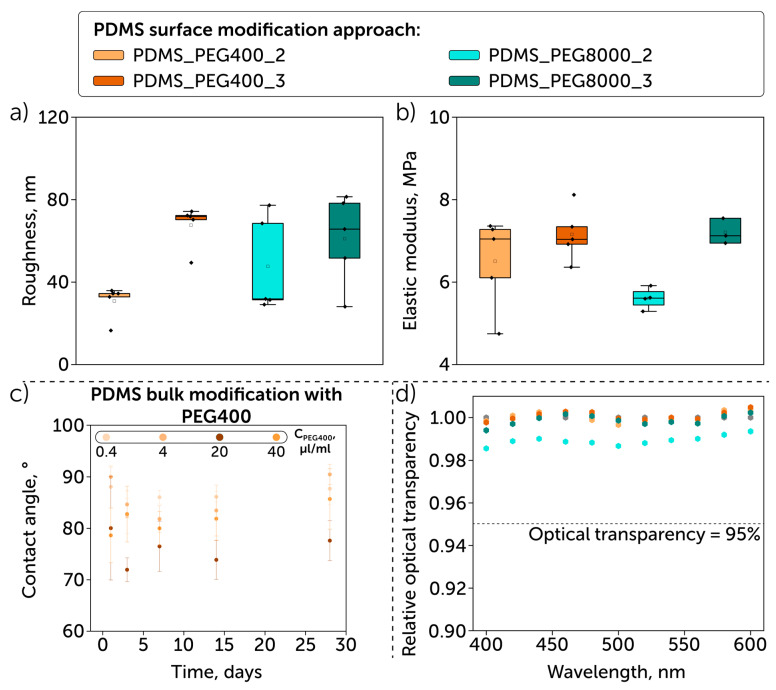
Mechanical properties of PEG-modified PDMS: (**a**) roughness; (**b**) elastic modulus; (**c**) hydrophilicity of bulk PEG-modified PDMS: while bulk modification ensured higher stability compared to surface grafting, the peak values were lower, which limits its potential for short-term applications; (**d**) optical transparency, which met the requirements for materials utilized for microfluidics and contact lenses fabrication.

We compared two PEG-based modifications: surface grafting and bulk modification. The second one, compared to the first one, showed higher stability for 4 weeks while having higher contact angle values (Figure 3c). We revealed the correlation between the PEG concentration and the contact angle value: the lowest values (20° at Day 3 and 30° at Day 28) were obtained when using a PEG concentration of 40 µL/mL, while the increase in its concentration led to the contact angle rise.

**Figure 4:**
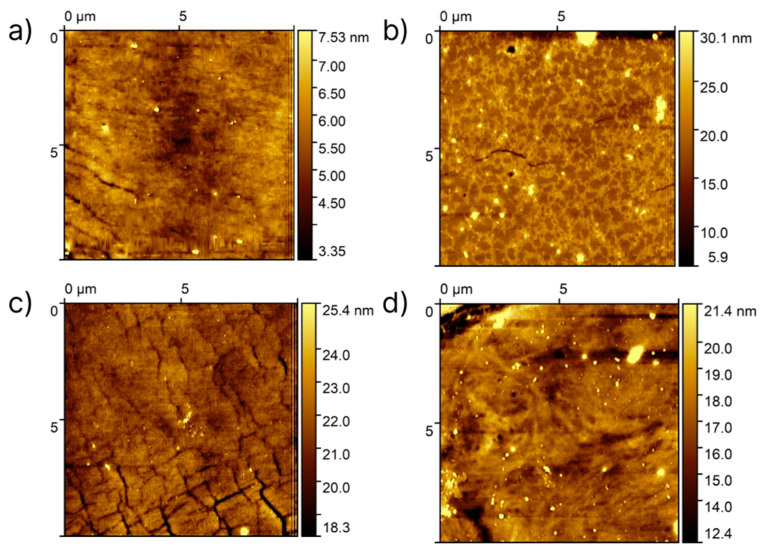
Surface characterization of PEG-modified PDMS by atomic force microscopy: (**a**) 400_2, (**b**) 400_3, (**c**) 8000_2, and (**d**) 8000_3.

The PDMS surface coating with PEG has a negligible effect on the roughness and stiffness measured. The samples’ roughness varied from 30.8 ± 8.0 nm for those treated with PEG400 (“grafting to”) to 61.0 ± 21.8 nm for those treated with PEG8000 (“grafting from”) (Figure 3a). A similar trend was shown for stiffness, which was maintained within a 5.6–7.2 MPa range (Figure 3b). Moreover, we studied the homogeneity of the PEG coating by atomic force microscopy, which showed the formation of a dense, uniform PEG layer on the surface of all studied samples (Figure 4 and Figure S5).

The relative optical transparency (relative transmittance) of PEG-coated PDMS samples is presented in Figure 3d. All samples exceeded the required range (95%), with the lowest relative transparency value of 98.6% (PDMS_PEG8000_2), while the others showed an insignificant difference compared to the non-modified PDMS (relative optical-transparency values for modified samples were normalized to the transparency of the non-modified sample).

**Figure 5:**
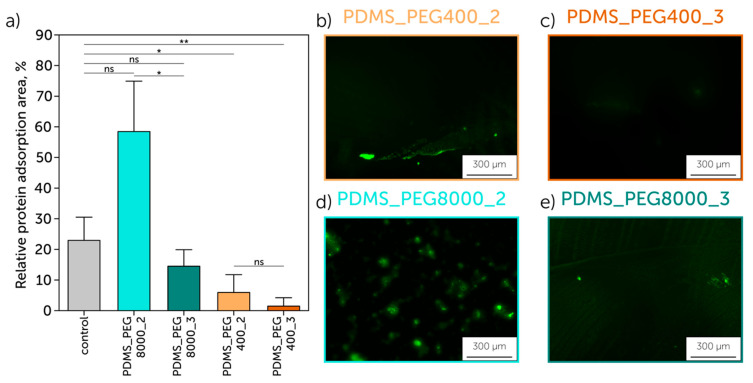
PDMS modified with PEG8000 exhibits a significant albumin adsorption, while using PEG400 results in the adsorbed protein absence: (**a**) relative protein adsorption area; (**b**–**e**) BSA-FITC conjugate adsorption on a samples’ surface: (**b**) PDMS modified with PEG400 using the “grafting to” method, (**c**) PDMS modified with PEG400 using the “grafting from” method, (**d**) PDMS modified with PEG8000 using the “grafting to” method, (**e**) PDMS modified with PEG8000 using the “grafting from” method. *p*-values: *, *p* < 0.05; **, *p* < 0.01; ns—not significant.

We analyzed the ability of the modified samples to possess antibiofouling properties. Initially, PDMS was shown to have a significant albumin adsorption (Figure S4). We revealed that PDMS modified with low molecular weight PEG (especially the “grafting from” method) demonstrated the absence of absorbed protein. In contrast, the coating with high molecular weight PEG had a negligible improvement compared to the non-modified PDMS (Figure 5b–e). The quantification of the adsorbed protein, calculated as the covered area degree, showed 58.5 ± 21.6% for PDMS_PEG8000_2, which was the highest value, while the modification with low molecular weight PEG (PDMS_PEG400_2) resulted in 5.9 ± 5.0%.

## 4. Discussion

The mechanism of the PDMS hydrophilization included the formation of a hydrophilic layer of PEG brushes. The presence and density of the PEG coating can be characterized via the elemental ratio calculations from XPS [32]. The increment in the C/Si ratio corresponded to the formation of a thicker coating. This increment correlated with the rise in the PEG molecular weight (the highest value (6.45) was observed for samples prepared with PEG8000). Further cross-linking via the plasma treatment was expected to densify the coating by the formation of a 2D network [33], resulting in the shrinkage of the layer thickness. While this effect was significant for PEG8000 coating, it was negligible for PEG400 due to the steric constraints of the short PEG chains bending, which partially prevented the formation of a network and kept more hydrophilic end groups on the material surface. AFM data also showed the formation of a homogeneous coating with all proposed approaches. Moreover, the obtained FTIR spectra showed the successful PEG coating deposition on a PDMS surface.

Despite the formation of a thicker layer while implementing PEG8000, these samples showed higher contact angles, being more hydrophobic compared to the coatings from low molecular weight PEG. This effect can be caused by a lower concentration of hydrophilic –OH end groups in high molecular weight PEG, while the –CH_2_–CH_2_– chain is a hydrophobic part of a coating. Moreover, cross-linking further eliminated –OH groups [8], leading to a slight worsening of hydrophilicity. In contrast, we revealed that the modification of the PDMS surface with low molecular weight PEG resulted in the formation of a more hydrophilic coating, which was even more stable due to the partial formation of a rigid (due to the short branches) 2D network [34]. The molecular weight range chosen allowed us to vary the contact angle values from 8.6 ± 3.5 to 16.7 ± 4.3°, while a further increase in PEG molecular weight (35 000 Da) was earlier shown to lead to higher values and lower stability (up to 60° at Day 18) [8]. Moreover, a recent study also reported the use of PDMS-PEG copolymers to reduce the PDMS hydrophobicity and showed results similar to those presented here [35]. When compared to the commercially applied method using poly(glycidyl methacrylate) and poly(acrylamide-co-acrylic acid), PEG grafting ensured significantly lower contact angle values and similar stability for one week [36]. The coatings formed using PEG-amines were less stable [24] as well; however, PEG-silanes were demonstrated to provide hydrophilization performance similar to that obtained here [37]. We also summarized the PEG-based PDMS modification approaches in Table 2.

In addition to the static conditions experiment, the evaluation of the contact angle retention under the perfusion conditions was performed to evaluate the applicability of the developed coatings for microfluidic applications [1]. While performing experiments under dynamic flow conditions (0.08 cm/s—capillaries and 4 cm/s—arteries [38]), we observed hydrophobicity recovery that could be caused by C–O–C bridges connected to PDMS surface groups. Thus, larger chains of high molecular weight PEG were less compact, therefore less stable under shear force [1]. Interestingly, the use of a higher flow rate resulted in lower contact angle values and higher coating stability. This can be explained by the orientation of polymer chains along the flow direction, thereby decreasing the surface entropy, which has been reported to enhance surface hydrophilicity [39]. The proposed modification showed more modest performance compared to zwitter-ionic silanes; however, it ensured more stable surface properties under static conditions [1].

Additionally, we tested bulk modification that was shown to enable the formation of a less hydrophilic surface with contact angles down to 70°. The lowest contact angle values were observed when we used a PEG concentration of 40 µL/mL. Increasing the concentration had the opposite effect and led to hydrophobicity recovery, apparently due to the phase delamination, presented as bubbles after washing out PEG during rinsing (Figure S6). An earlier conducted study on bulk modification showed that only the use of polyethylene oxide (PEO, generally referred to as PEGs with molecular weight higher than 20 kDa) instead of PEG led to low contact angle values (up to 20°), apparently due to the inhibited phase delamination, which was caused by slower diffusion of long PEO chains [40].

As roughness may significantly affect the laminar flow behavior according to the Colebrook–White equation and impair the microfluidic system performance [41,42,43,44], we assessed this parameter. The data obtained showed it was up to 80 nm with insignificant difference among the approaches described above (Figure 3a); therefore, it does not influence the flow behavior significantly. The PDMS stiffness is especially important for contact lenses [45] and microfluidic organ-on-a-chip systems [46]. Here, we focused on retaining the initial value, which can be further tailored via various approaches [47]. We revealed that the proposed approaches had no significant influence on stiffness (except for using the “grafting from” method and PEG8000). This effect can be explained by the presence of a soft layer of randomly oriented PEG brushes.

Another parameter is optical transparency, which should be no lower than 95% [48], e.g., for contact lenses. All samples tested met this requirement and showed the relative transparency values close to 100%. Interestingly, the second plasma treatment of samples covered with PEG8000 ensured an increase in their value: from 98.6% to 100%. Compared to bulk modification and implementation of the PDMS-PEG copolymers, PDMS grafting with PEG demonstrated high optical transparency while preserving similar contact angles [35,40].

Furthermore, PEG-based modification of the PDMS surface was implemented to address the issue of non-specific protein adsorption, which may affect the bioresponse of devices and drug adsorption in drug screening [35]. The hydrophilic coatings were found to ensure a lower protein adsorption compared to hydrophobic surfaces [49]. In contrast, hydrogels tend to swell significantly, causing an additional protein uptake [50]. PEG400-modified PDMS showed the highest antibiofouling efficiency (down to 5.9% of relative protein adsorption area for the “grafting to” method), especially when combined with the “grafting from” approach (almost no adsorbed protein detected—1.5% of relative protein adsorption area). This effect arose from the low swelling ratio for short PEG400 chains. Similar performance with low adsorption was shown for PDMS-PEG copolymer [35] and low molecular weight (575 Da) PEG [51], while the application of PEG with the molecular weight of 5000 Da showed a significant protein uptake [52], similar to the results obtained in this paper. The main possible reason for a significant protein adsorption by PEG8000-formed coatings is swelling of PEG brushes due to the high chain length, which can entrap protein molecules. This hypothesis is supported by lower protein uptake when using the second plasma treatment, which causes cross-linking of PEG brushes.

## 5. Conclusions

In our study, we have proven that the use of low molecular weight PEG ensured better surface hydrophilization (the lowest detected contact angle was 8.6 ± 3.5°) and stability under dynamic flow conditions. The coating with high molecular weight PEG was denser and thicker; however, this led to increased protein uptake. In contrast, low molecular weight PEG enabled the antifouling effect of the treated surface. The applied modification approaches showed no significant effect on roughness and elastic modulus while keeping the optical transparency. Thus, the “grafting from” approach using low molecular weight PEG can be considered optimal for forming hydrophilic coatings on a PDMS surface. Despite the significant hydrophilization accompanied by the preservation of mechanical properties, the operational stability still needs further improvement, since the hydrophobicity recovery was observed within 1–3 days.

## Figures and Tables

**Table 1:** Elemental concentrations (XPS analysis) represent the growth of the C/Si ratio for PDMS samples coated with high-molecular-weight PEG).

Sample	Element Concentration, %			C/Si Ratio
	C	O	Si	
PDMS (non-modified) ^a^	41.2	35.3	23.2	1.77
PDMS_PEG400_2	33.1	43.6	23.3	1.42
PDMS_PEG400_3	35.9	42.4	21.7	1.65
PDMS_PEG8000_2	59.6	31.2	9.2	6.45
PDMS_PEG8000_3	43.1	38.4	18.5	2.33

^a^ the sum of the concentrations for the pristine PDMS sample is not equal to 100, since trace elements were detected (overall concentration—0.3), which were considered not to represent the effect of PEG coating (Na).

**Table 2:** Comparison of the hydrophilization efficiency between different coatings.

Coating Composition	Contact Angle, ° ^1^	Stability of Coating, Hours ^2^	Reference
PEG35000	46	20	[8]
PEG-amine	70	–	[25]
PEG-silane	~10	24	[33]
Poly(glycidyl methacrylate) and poly(acrylamide-co-acrylic acid)	25–35	>168	[33]
PEG400	10–20	>500	This study
PEG8000	10–30	>500	This study

^1^ measured at Day 1; ^2^ estimated as the time of CA recovery to 40°.

## Data Availability

The original contributions presented in this study are included in the article/supplementary material. Further inquiries can be directed to the corresponding author.

## Supplementary Materials

The following supporting information can be downloaded at https://www.mdpi.com/article/10.3390/polym17243296/s1: Figure S1: Survey XPS spectra of the PEG-modified PDMS: (a) PEG400_2, (b) PEG400_3, (c) PEG8000_2, (d) PEG8000_3, (c) initial PDMS; Figure S2: XPS spectra of the initial PDMS: (a) Si2p, (b) O1s; Figure S3: FTIR spectra of PEG800-modified PDMS; Figure S4: Albumin adsorption on the surface of the initial (non-modified) PDMS; Figure S5: AFM data of raw PDMS; Figure S6: SEM images representing the phase delamination that occurred during the bulk PEG-based modification of PDMS. Table S1: Approximation parameters for the dependence of the contact angle on time in a dynamic system (capillary flow); Table S2: Approximation parameters for the dependence of the contact angle on time in a dynamic system (arterial flow).

## Abbreviations

The following abbreviations are used in this manuscript:

PDMS	Polydimethylsiloxane
PEG	Polyethylene glycol
XPS	X-ray photoelectron spectroscopy
AFM	Atomic force microscopy
BSA	Bovine serum albumin
FITC	Fluorescein isothiocyanate isomer I
