# Supplementary material for: Tuning Poly(dimethylsiloxane) Hydrophilization and Coating Stability via the Optimization of Polyethylene Glycol Molecular Weight
Source: Polymers (Basel). 2025 Dec 12;17(24):3296. doi: 10.3390/polym17243296 (PMC12736552; doi:10.3390/polym17243296)
Supplement: Supplementary file 1 [file polymers-17-03296-s001.zip › polymers-4008887-supplementary.pdf]

Supporting materials

Tuning Poly(dimethylsiloxane) Hydrophilization and Coating Stability via the Optimization of Polyethylene Glycol Molecular Weight

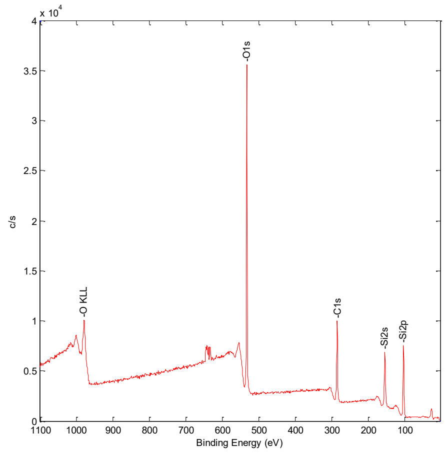

a

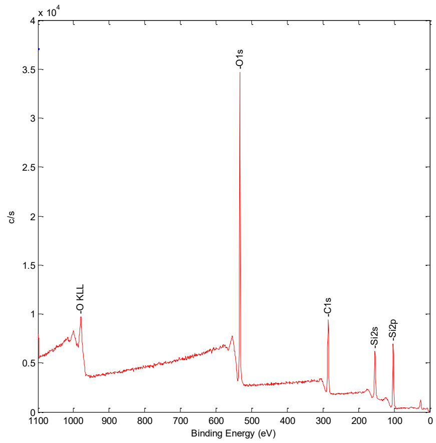

b

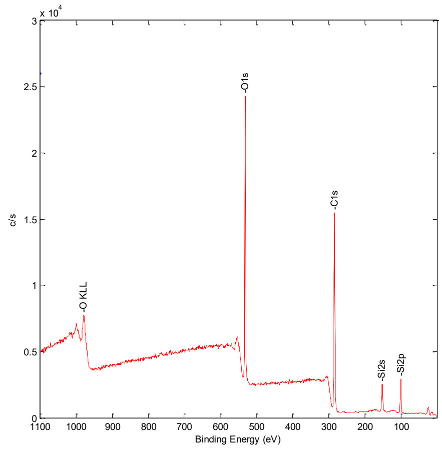

c

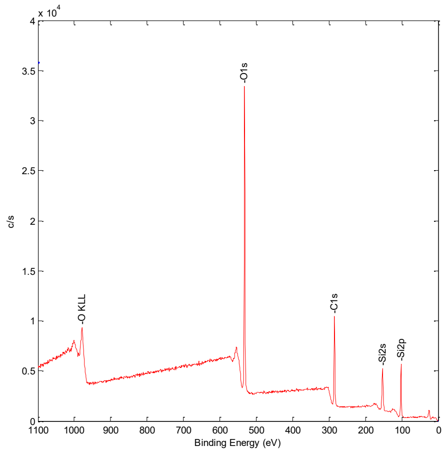

d

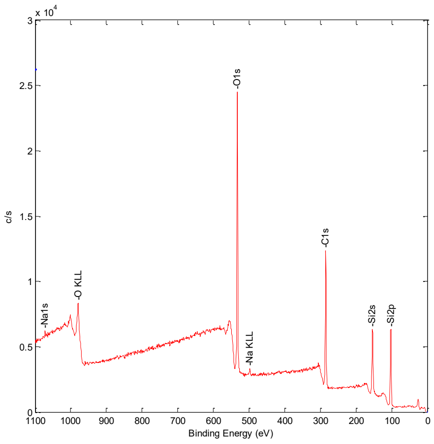

e

Figure S1. Survey XPS spectra of the PEG-modified PDMS: a) PEG400_2, b) PEG400_3, c) PEG8000_2, d) PEG8000_3, c) initial PDMS.

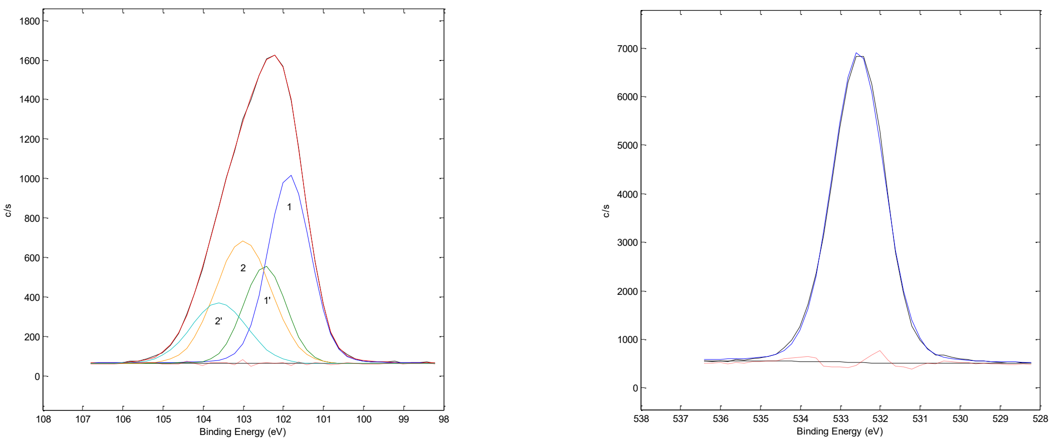

a

b

Figure S2. XPS spectra of the initial PDMS: a) Si2p, b) O1s

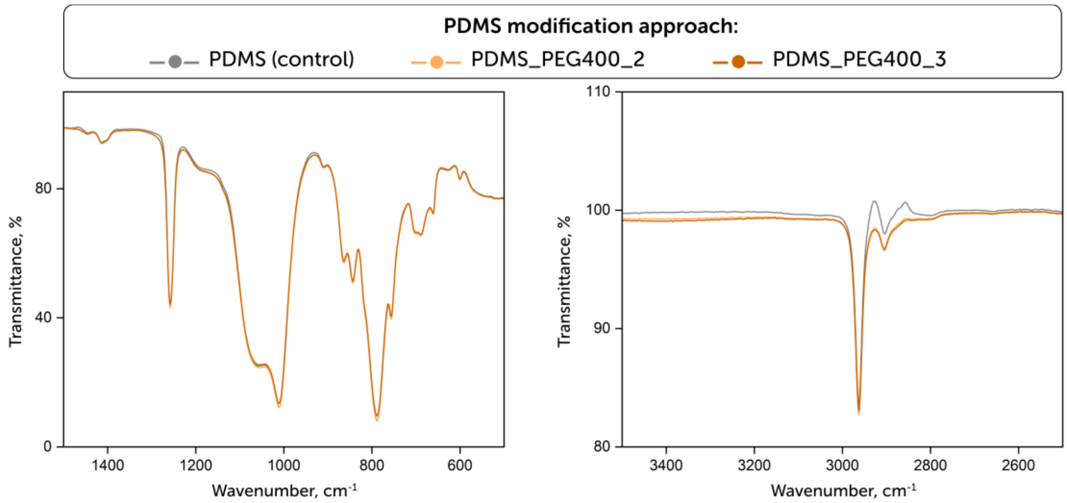

Figure S3. FTIR spectra of PEG800-modified PDMS

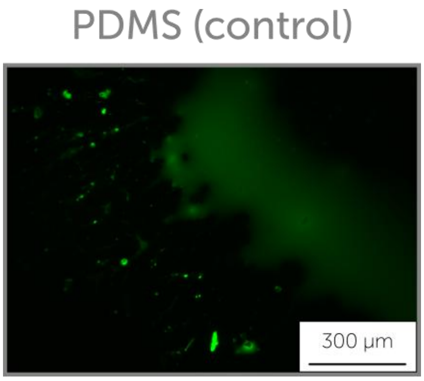

Figure S4. Albumin adsorption on the surface of the initial (non-modified) PDMS.

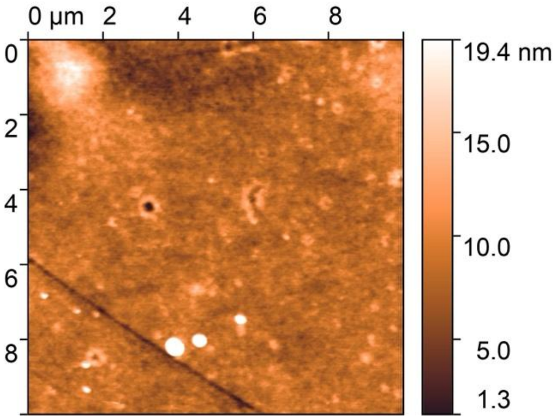

Figure S5. AFM data of raw PDMS.

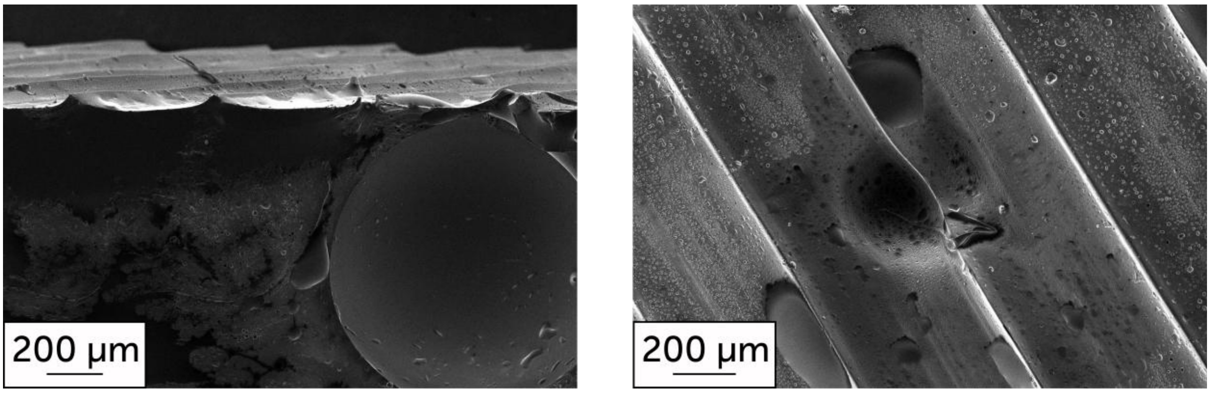

Figure S6. SEM images representing the phase delamination occurred during the bulk PEG-based modification of PDMS.

Table S1. Approximation parameters for the dependence of the contact angle on time in a dynamic system (capillary flow)

**Modification approach**	**Y = I + B1*x + B2*x^2**		
	**I**	**B1**	**B2**
**PEG400_2**	**20.3 ± 6.0**	**27.6 ± 9.7**	**– 5.8 ±3.1**
**PEG400_3**	**29.6 ± 1.4**	**18.8 ± 2.3**	**– 4.5 ± 0.7**
**PEG8000_2**	**13.4 ± 2.1**	**52.6 ± 3.4**	**– 13.9 ± 1.1**
**PEG8000_3**	**15.3 ± 0.2**	**38.7 ± 0.3**	**– 9.3 ± 0.1**

Table S2. Approximation parameters for the dependence of the contact angle on time in a dynamic system (arterial flow)

**Modification approach**	**Y = A + B*x**	
	**A**	**B**
**PEG400_2**	**27.8 ± 3.2**	**11.6 ± 1.7**
**PEG400_3**	**30.0 ± 0.9**	**8.5 ± 0.5**
**PEG8000_2**	**11.4 ± 4.7**	**13.2 ± 2.5**
**PEG8000_3**	**12.0 ± 6.1**	**12.8 ± 3.2**
